# Exosomes as mediators of signal transmitters in biotoxins toxicity: a comprehensive review

**DOI:** 10.1007/s10565-024-09867-4

**Published:** 2024-05-02

**Authors:** Tongxiao Xu, Bingxin Huangfu, Xiaoyun He, Kunlun Huang

**Affiliations:** 1 Key Laboratory of Precision Nutrition and Food Quality, Key Laboratory of Functional Dairy, Ministry of Education; College of Food Science and Nutritional Engineering; China Agricultural University, Beijing, 100083 China; 2 Key Laboratory of Safety Assessment of Genetically Modified Organism (Food Safety), Ministry of Agriculture and Rural Affairs of the People’s Republic of China, Beijing, 100083 China

**Keywords:** Biotoxin, Exosome, Delivery, Immunity

## Abstract

Small membranes known as exosomes surround them and are released by several cell types both in *vitro* and in *vivo*. These membranes are packed with a variety of biomolecules, including proteins, lipids, deoxyribonucleic acid (DNA), ribonucleic acid (RNA), and non-coding RNA (ncRNA). As a source of biological nanomaterials, exosomes play a role in information and substance transmission between cells and have been identified as a general method of facilitating communication during interactions between the body, target organs, and toxins.. In order to understand the changes and mechanism of the composition and level of exosomes after biotoxin infection, this review focuses on current findings on the exosomes and highlights their novel uses in the toxicity mechanism. Exosomes are mainly used as a delivery carrier or mediated by receptors, and play an immune role after the toxin enters the body. This review expounds on the importance of exosomes in the toxicological mechanism of biotoxins and provides new insights for further diagnosis of toxic biomarkers, detoxification, and treatment development.

**Graphical abstract:**

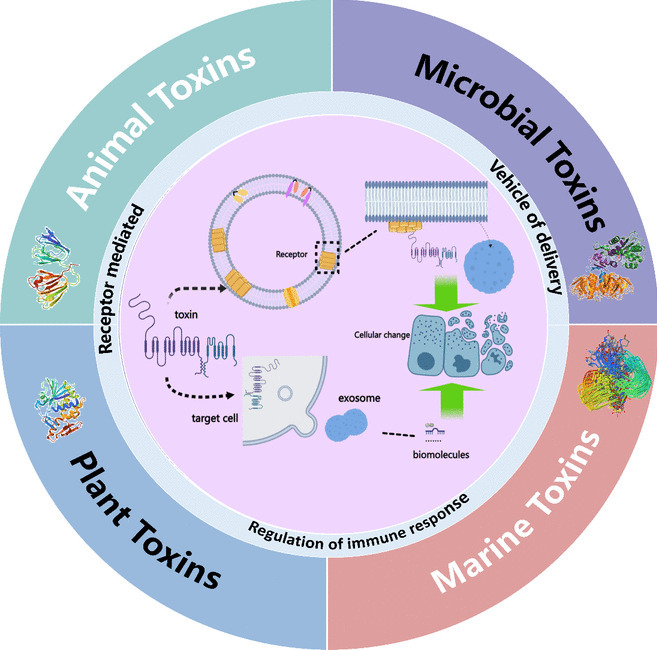

## Introduction

Exosomes are membranous vesicles released into the extracellular matrix after the fusion of intracellular poly vesicles and cell membranes (Kalluri and LeBleu 2020). Almost all types of cells produce and release exosomes. Exosomes are nano-scale lipid inclusion structures with a diameter of 30–150 nm, which are coated with lipids protein, DNA, RNA and ncRNA. Body fluids like blood, saliva, urine, and breast milk all contain exosomes, where they travel through the circulatory system to reach other cells and tissues, allowing for remote regulation (Zhang et al. 2015; Kalluri and LeBleu 2020).

Toxic natural substances (such as zearalenone, vomitoxin), also known as biotoxins, contaminate food, pet food, and livestock feed, posing risks to the environment and feed chain (including the aquaculture industry). Biotoxins are non-replicable toxic chemicals by living cells for self-defense (Xu et al. 2021). Although much effort has been invested in toxin research, the control and toxicological assessment of toxic secondary metabolites still face many problems. The agriculture, food, and feed industries are vulnerable to biotoxin contamination. In addition, studies on mechanisms of toxicity, especially chronic toxicity, remain insufficient. In more detail, the biotoxins' toxic mechanisms refer to their action patterns, toxicokinetics, adverse effects in humans and animals, including the combined effects of mixtures, and the interaction of these toxin mixtures at the organ and cellular level (Kordiš and Gubenšek 2000). Therefore, more research is required to completely understand the harmful consequences of biotoxins in the environment and food chain. more research is needed to fully comprehend the toxic effects of biotoxins in the environment and feed chain. Biotoxins typically exhibit a strong selective action on cell membranes, ion channels, receptors, ribosomal proteins, and other specific target molecules that participate in living systems and metabolic processes, resulting in varying degrees of lethal or toxic effects (Ménez 1998; Kordiš and Gubenšek 2000; Yuan et al. 2022).

Exosomes can cross biological barriers and participate in short and long-distance intercellular communication (He et al. 2018). However, there are no specific and validated biomarkers and mechanisms explaining how most biotoxins, such as mycotoxins, exert their effects after entering the body. In addition, exosomes act as biomarkers to provide unique information about the comprehensive toxicity of low-dose toxin mixtures. This makes it possible to accurately assess the health risks of exposure to biotoxin mixtures for both humans and animals. Based on existing research, this review aims to identify the toxic mechanism of biotoxins and summarize the research progress of exocrine in the toxicological mechanism of biotoxins, providing theoretical references for their toxicity mechanism, detoxification, and therapies.

## Exosome

Exosomes are double-layer vesicles with a diameter of about 30-150 nm secreted by cells, which are packed with a large number of bioactive substances such as proteins, microRNA (miRNA), and long noncoding RNA (lncRNA) (Zhang et al. 2015; Fuchs et al. 2021). The exosomes widely exist in blood, urine, cerebrospinal fluid, saliva, and milk (Hariharan et al. 2021). Most eukaryotic cells secrete exosomes into the extracellular space, and these exosomes are vesicles that promote intercellular signal communication at the molecular and physiological levels (Cun et al. 2022).

As a new type of extracellular vesicles, exosomes are transported between cells by surface ligands or between cells. They are directly or indirectly involved in intercellular communication (David and Zimmermann 2020). When the external environment stimulates the body, corresponding tissue cells receive signals that either increase exosome production or inhibit exosome secretion. When exosomes are discharged and released outside the cell (David and Zimmermann 2020), they selectively package proteins, nucleic acids, cytokines, and other active substances from the donor cells. The contents are then released upon entering the recipient cells. Therefore, it can cause functional responses and alter recipient cell phenotypes, affecting their physiological states. Exosomes are associated with immune response, viral pathogenicity, pregnancy, cardiovascular disease, central nervous system-related diseases, and cancer (Doyle and Wang 2019). The proteins, metabolites, and nucleic acids delivered by exosomes to recipient cells can effectively alter their biological responses. This exosome-mediated response can exacerbate or alleviate disease (Hamzah et al. 2021). The intrinsic properties of exosomes in regulating complex intracellular pathways enhance their potential utility in the therapeutic potential of many diseases.

### Engineering exosomes

**Fig. 1:**
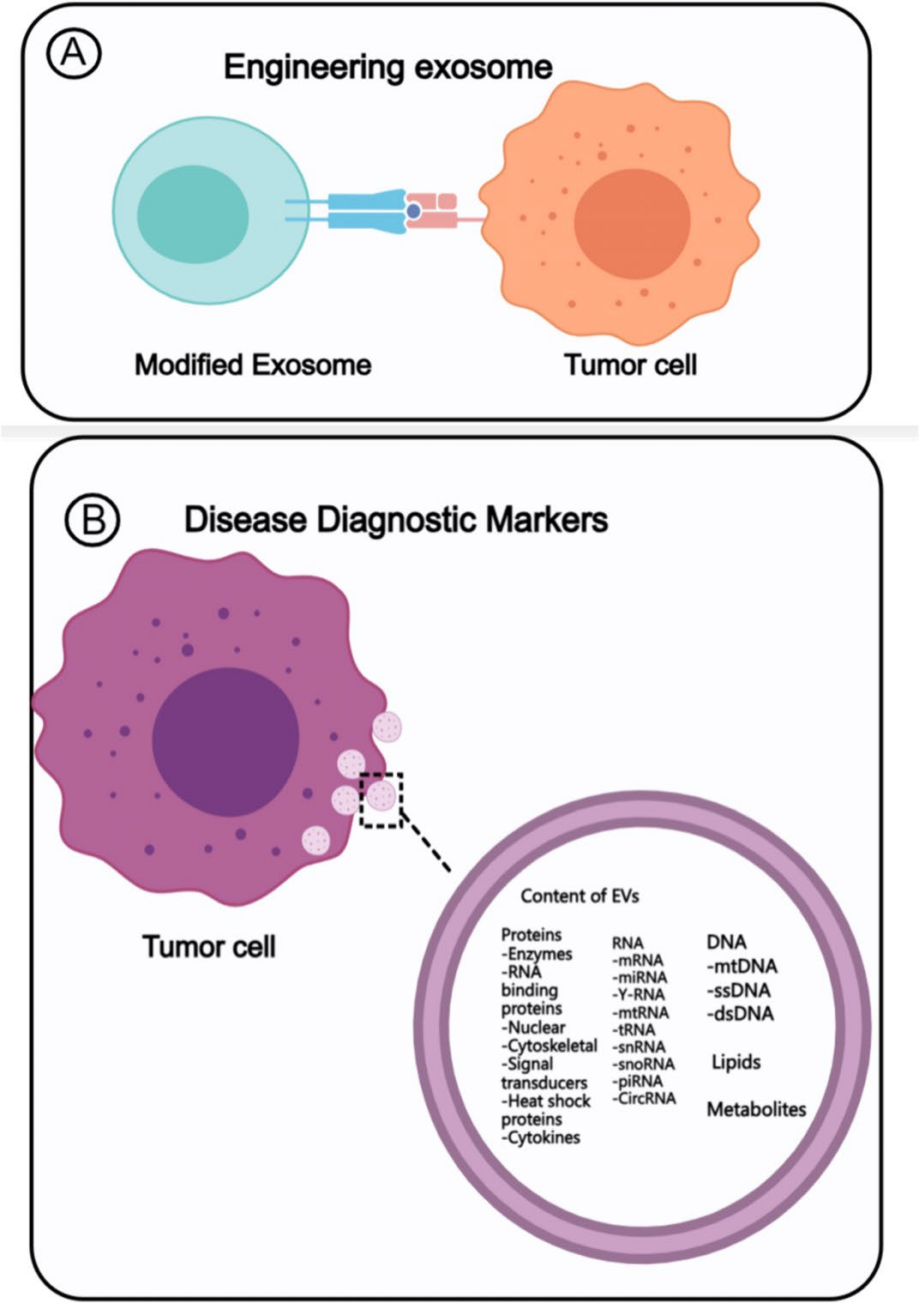
The application of exosomes. **A**. Engineered exosomes to deliver drugs to treat tumor cells. **B**. Exosomes serve as disease markers

Exosomes have been investigated for use as drug delivery systems for a variety of diseases due to their low immunogenicity, preference for tumor localization, endogenous and acquired targeting, and stability (Fig. 1) (Zhang et al. 2019; Shao et al. 2020). At present, most of the engineering exosomes on the market are modified by milk-derived and HEK293 cell-derived exosomes, and the exosomes of these two sources are relatively stable. Some hydrophobic or hydrophilic small-molecule drugs can be delivered to targeted locations by loading into exosomes (Yang et al. 2019; Hade et al. 2021). In general, exosomes can act as a delivery vehicle for small molecules, ultimately leading to a higher accumulation of the molecules in target cells, increasing the existence time of drugs in the blood circulation, improving the stability of small molecules, and thus increasing the therapeutic efficiency of small molecule drugs (Zhao et al. 2022). Duan et al. (Duan et al. 2021) used exosomes to encapsulate curcumin and delivered it to microglia by nasal delivery. Curcumin-coated exosomes were shown to be protective against lipopolysaccharide (LPS)-induced encephalitis, experimental autoimmune encephalitis, and inhibited myelin oligodendrocyte glycoprotein-induced experimental autoimmune encephalomyelitis (Gurung et al. 2021). Plant organs also secrete exosomes (plant exosome-like nanovesicles, PELNVs), and plants are a cost-effective source of exosomes with high production performance (Zhang et al. 2018). Generally, they are extracted from edible plants, can be efficiently produced in large quantities, and can compensate for the limitations of mammal-derived exosomes, which have attracted more and more attention (Cao et al. 2023). At present, there are exosomes from grape (Teng et al. 2022), broccoli (Duan et al. 2023), ginger (Zhu and He 2023), carrot (Mu et al. 2014), and other sources (Record 2013), which are similar to general animal derived exosomes in terms of size distribution, morphology, and contents. They also have certain clinical applications. For example, Ju et al. (Ju et al. 2013) demonstrated that grapevine exosome-like nanoparticles (GELNs) could target intestinal stem cells. GELNs could penetrate the mouse intestinal mucus barrier and be taken up by intestinal stem cells. Lgr5hi intestinal stem cells were induced by the Wnt/β-catenin pathway. GELN treatment resulted in activation of the Wnt-mediated Tcf4 transcriptional machinery in the crypts, and regulates the proliferation of stem cells. The most widely used ginger exosomes have been verified to deliver anticancer drugs and inhibit tumor growth (Brahmbhatt et al. 2013). At the same time, Given exosomes extracted from ginger caused changes in intestinal miRNAs in mice with colitis. Combined analysis of intestinal flora showed that miRNAs regulated the level of intestinal microorganisms, thereby normalizing impaired intestinal function (Zhang et al. 2016).

### Exosomes as diagnostic markers for disease

As exosomes reflect some of the characteristics of parental cells, exosomes secreted by tumor cells indicate the features of parental tumor cells (Fig. 1). Thus, analyzing the expression of exosome protein or RNA is a powerful tool for disease diagnosis (He et al. 2018). Exosomes improved the apoptosis of intestinal epithelial cells induced by vomitoxin (DON) and reversed the inhibitory effect of DON on the proliferation of intestinal epithelial cells. Further exploration revealed that miR221 and miR222 in intestinal epithelial cells were associated with apoptosis and proliferation of intestinal epithelial cells through the regulation of phosphatase with tensor protein homolog (PTEN) as a target gene. Moreover, the apoptosis and proliferation inhibition of intestinal epithelial cells induced by DON depends on PTEN, whereas miR221 and miR222 could improve intestinal epithelial cell damage by targeting PTEN (Khayambashi et al. 2021). In prostate cancer, two different exosome biomarkers, prostate cancer-associated 3 (PCA-3) and transmembrane serine protease 2 (TMPRSS2), are directly associated with incidence. Proteins derived from urine exosomes are diagnostic markers for urinary tract diseases and bladder cancer (Khayambashi et al. 2021). In various studies, exosome miRNAs have been shown to serve as biomarkers to diagnose renal fibrosis and cardiovascular diseases (Li et al. 2021). Compared with healthy individuals, the content of serum exosomes is slightly upregulated in patients with benign tumors while significantly increased in cancer patients (Liang et al. 2021). There are about 175 miRNAs commonly found in both tumor cells and exosomes, and the raised miRNAs in exosomes are distributed. The distribution of up-regulated miRNAs in exosomes matches the distribution of up-regulated miRNAs in ovarian cancer patients at different stages, but this method cannot completely distinguish different stages of cancer (Liu et al. 2018; Qiu et al. 2022). Therefore, it is crucial to develop relevant technologies that can enrich disease-related (such as tumor-derived) exosomes and identify their specific components, thereby significantly improving the sensitivity of exosomes as biomarkers (Sun et al. 2018a).

## Biotoxins

**Fig. 2:**
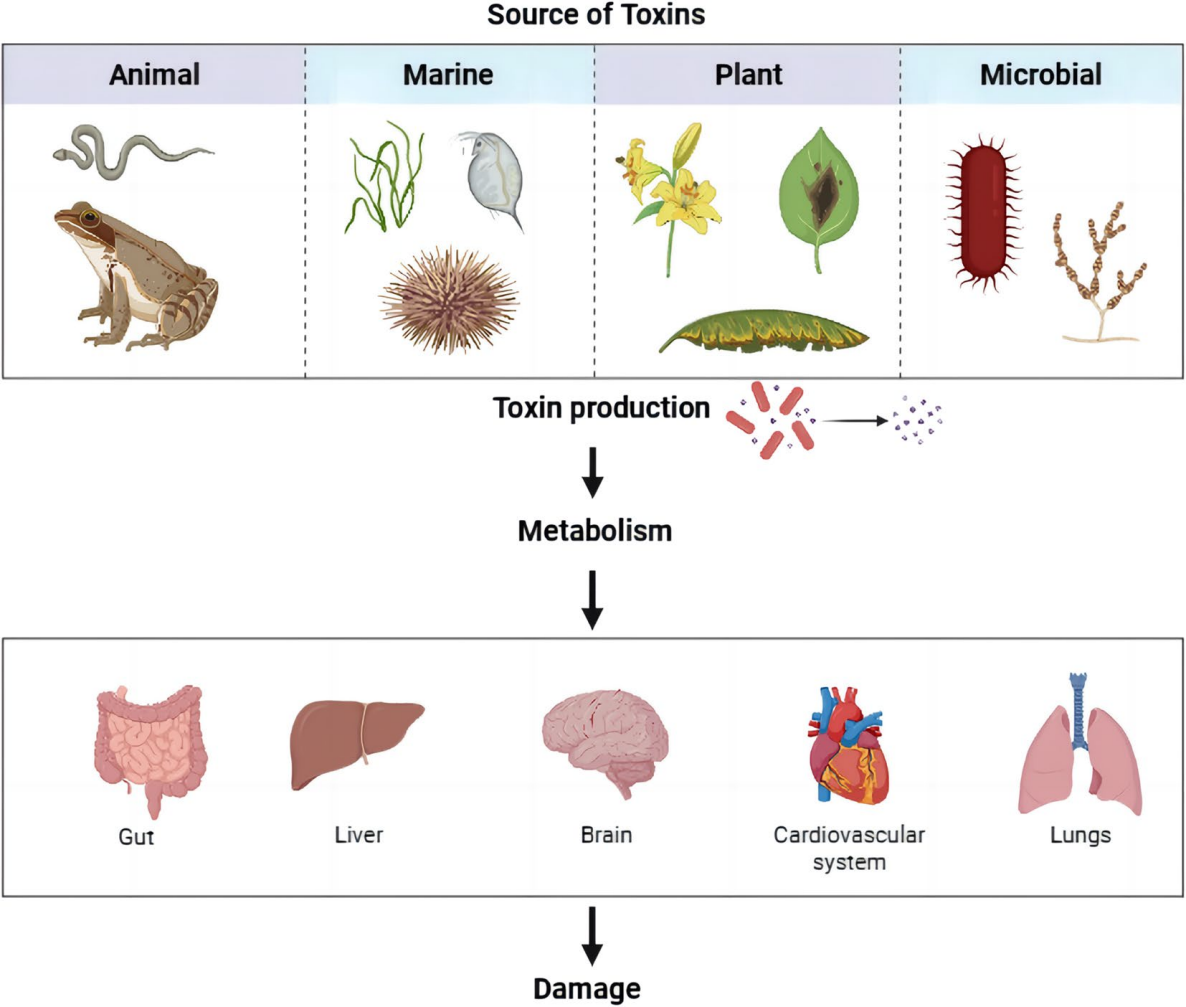
Sources and hazards of biotoxins. Biotoxins can be divided into animal toxins, Marine toxins, phytotoxins and microbial toxins. After toxins are produced, they enter the body and are metabolized, which can cause different degrees of damage to different organs

Biotoxins, also known as biological toxins and natural toxins, are metabolites derived from various organisms (Clark et al. 2019; Panda et al. 2022). These harmful biological agents cannot reproduce within organisms and pose threats to other species. Due to the diversity of biological species, their corresponding metabolites, biotoxins, show diverse characteristics, such as source, chemical structure, and mechanism of action (Herzig et al. 2020). Some of them can cause ignorable toxic effects on biological organisms. Therefore, It is crucial to study the involvement of exosomes in the toxicity mechanisms of biotoxins. Biotoxins can be classified into animal toxins, marine toxins, phytotoxins, and microbial toxins based on their origin (Fig. 2).

### Animal toxins

The evolution of animal toxins is closely inspired by the natural world. To protect themselves and hunt for prey, some terrestrial animal species secrete toxins and interfere with or destroy the physiological processes of other organisms through bioactive substances such as toxins themselves and gene sequences, thus affecting the natural environment, affecting the natural environment, and pollute food and livestock feed through the food chain, and then harming human health (Zhang 2015; Kozlov 2018; Yu et al. 2020). Animal biotoxins are primarily produced by the venomous glands of venomous animals (Zhao et al. 2019). Most of them are protein compounds with peptide bonds and disulfide bonds (Kordiš and Gubenšek 2000). They target specific organisms through protein sites or ion channels by binding to receptors and play their role in disrupting physiological processes (Ménez 1998; Yuan et al. 2022), resulting in hematologic toxicity, neurotoxicity, and cytotoxicity. Common animal toxins include hydrocarbons, heterocyclic compounds, alkaloids, biogenic amines, peptides, and proteins, such as snake venom, bee venom, and scorpion venom (Sukumwang and Umezawa 2013; Undheim et al. 2016; Waheed et al. 2017; Walker 2020; Johannes 2022).

### Marine toxins

Marine toxins are different from animal toxins. Marine biotoxins refer specifically to the toxic metabolism of marine organisms, which include algae, shellfish, coelomatids, and some tropical coral reef fish (Salzman et al. 2006). They are the most widely distributed and most toxic biotoxins (Halstead 1981). Marine biotoxins can be divided into three categories based on their chemical structures: peptide toxoids, polyether toxoids, and alkaloid toxoids. Marine toxins exhibit high selectivity for receptors. They typically act on nerves and muscles to stimulate specific targets on cell membranes, such as neuroreceptors or ion channels, thereby affecting a range of receptor-related cellular regulatory activities.(Van Dolah 2000; Leal and Cristiano 2022). Also, they exhibit a wide range of toxicity in the nervous, cardiovascular, and cytotoxic systems (Ye et al. 2019).

### Phytotoxins

Competitive evolution between plants and herbivores forced plants to produce a wide variety of toxic secondary metabolites to protect themselves, and these secondary metabolites are known as phytotoxins (Strobel 1982; Adamski et al. 2021). However, phytotoxins inhibit plant growth and harm herbivores and humans above the maximum tolerated doses (Bucheli 2014). Phytotoxins have no common structural characteristics, and they come in different categories, such as non-protein amino acids, peptides, proteins, alkaloids, and glycosides or combinations of these categories (Eberhard and Ulrike). Aconitoid alkaloids, brucine, and muscarine are common plant toxins. When herbivores consume phytotoxins, the toxins are transported to their gastrointestinal tract, attack cells, and inhibit critical cell function (Finkelstein et al. 2010). However, the animal's body itself catalyzes the degradation of toxins through specific enzymes to alter their molecular structure, such as malonylation of phenolic glycosides, thereby reducing their toxicity. Also, some hydrolases can degrade the activity of enzymes and interact with each other to chelate toxic secondary metabolites produced by plants (Boeckler et al. 2011).

### Microbial toxins

The vast majority of foodborne diseases result from microbial toxins, which are produced during microbial growth and reproduction. They are secondary toxic biotoxins second only to marine toxins. According to sources, they can be classfied into two categories: mycotoxins and bacterial toxins (Schmitt et al. 1999; Pleadin et al. 2019; Xiong et al. 2022). In more detail, common mycotoxins include aflatoxin, ochratoxin, and zearalenone (Alshannaq and Yu 2017). Bacterial toxins are generally two-component protein toxins and LPS endotoxins, such as botulinum toxin, cholera toxin, and enterotoxin (Faïs et al. 2018; Lemichez et al. 2020). Animal tissues, such as the liver, kidney, or gastrointestinal tract, can metabolize these toxins and generate secondary toxic metabolites, leading to liver and kidney toxicity and gastrointestinal dysfunction (Pier 1992; Alshannaq and Yu 2017). Therefore, attention is paid not only to the toxins themselves but also to their metabolite in the study of microbial toxins (Stone and Darlington 2017).

The toxic effects of biotoxins work through various mechanisms, such as inducing oxidative damage and apoptosis, and exosomes usually transmit signals through this general pathway. Moreover, oxidative stress is the production of large amounts of free radicals, such as reactive oxygen species (ROS) by the body, leading to a variety of harmful stimuli. Untimely free radical scavenging leads to the imbalance of oxidative and antioxidant systems in the body and causes oxidative damage to tissues and cells (Lai et al. 2022). As one of the initial signals produced after cell stress, ROS is critical in the toxic mechanism of biotoxins by lipid peroxidation, reducing the activity of antioxidant enzymes and inducing DNA damage (Liang et al. 2021). In contrast, intracellular ROS increases the number of MVBs by inhibiting their degradation in lysosomes, thereby enhancing the release of exosomes under the synergistic effect of glutathione (GSH) depletion on the external surface that can be regulated by oxidative stress (Liu et al. 2018). Higher ROS levels can reduce the production of exosomes by activating cell autophagy to degrade MVBs. Hypoxia-inducible factor (HIF) and exosomes are involved in the immunotoxicity of trichothecenes (T-2 toxin). T-2 toxin triggers cellular hypoxia, while mitochondria produce abundant ROS under hypoxic conditions and activate Hypoxia-inducible factor 1 subunit alpha (HIF-1α) signaling. The release of exosomes is related to HIF-1α and controls the adaptive physiological response to hypoxia through the NF-κB pathway (Wu et al. 2020). In addition, Milane et al. (Milane et al. 2015) demonstrated that the regulation of exosome release is dependent on the GSH level on the external surface regulated by oxidative stress. Likewise, cigarette smoke extract (CSE) caused oxidative stress and enhanced the release of exosomes from human bronchial epithelial cells (Noonin and Thongboonkerd 2021).

The mechanism of toxin-induced cellular exosomes is unique from the others. For instance, OTA-induced cytotoxicity is partly delivered by EXO-OTA. This exosome leads to cell cycle arrest and alters protein metabolism, resulting in cytotoxicity, which can further worsen developmental delays and other diseases (Zhu et al. 2021). Toxin-treated nasopharyngeal carcinoma cells produce exosomes containing latent membrane protein-1 (LMP1). Consequently, neighboring cells uptake these exosomes and alter their transcriptional profile (Yang et al. 2019). The transcriptional changes through LMP1-mediated release of epidermal growth factor receptor from exosomes further result in the activation of ERK and PI3K/Akt pathways in epithelial cells, endothelial cells, and fibroblasts. These pathways are well-known for promoting cell growth and migration (Yang et al. 2017). After the body is exposed to the toxin, the secretion of exosome often increased or decreased. The most studied is that the level of miRNA contained in exosomes will also increase or decrease accordingly, and then cause changes in the genetic environment. In addition, exosomes can also contain toxins under special circumstances, which can cause further cytotoxicity. For example, exosomes released from Stx2a-treated human macrophage like cells are even more cytotoxic to HK-2 cells than toxin exposure alone. In addition, some exosomes as a key part of oxidative stress play a role through the expression level of the contained substances, affecting the body's resistance to toxins, or further deepening the damage of the body.

## Exosomes as biotoxin toxicity signal transmitters

Biotoxins disrupt intercellular signal transduction, induce oxidative damage and apoptosis, and exhibit high toxicity to the kidney, liver, nerve, and reproductive systems (Yang et al. 2018; Liu et al. 2021; Huang et al. 2022). Therefore, exosomes significantly contribute to the toxic process of biotoxins. At the molecular level, the expression of exosomes on receptor cells and cell surface receptors varies according to the toxin type and target cells (Table 1). Existing studies found that this functional heterogeneity enables the exosomes to induce cell survival, apoptosis, or immune regulation. All these features result in a higher level of complexity and heterogeneity in the toxic effects of biotoxins (Joyce et al. 2016).

**Table 1:** Mechanisms of exosome-mediated biotoxin toxicity

	Toxin Type	The Role of Exosome	Mechanism of toxin toxicity mediated by exosome	Reference
Animal Toxins	β γ-CAT isolated from B.maxima skin secretions	Regulation of immune response	Stimulate mice to produce functional exosomes and activate immune T cell response	(Deng et al. 2020)
Plant Toxins	TCS	Vector of delivery	TCS uses the delivery of exosomes to form unique toxin-loaded vesicles	(Zhang et al. 2009)
Microbial Toxins	Endotoxin	Vector of delivery	The exosomes containing miR-146a and miR-155 were delivered to the target organs to inhibit the changes of target genes and inflammatory response in vivo	(Alexander et al. 2015)
	Endotoxin	Vector of delivery	Overexpression of exosome circANTXR1 promotes the proliferation and metastasis of HCCLM3 cells	(Huang et al. 2021)
	T-2 toxin	Receptor-mediated	The exosome was used as a safe transport carrier for receptor cells to transmit HIF-1 α through the exosome. The release of exosomes is related to HIF-1 α in hypoxic tumors, which is beneficial to immune escape	(Wu et al. 2020)
	CT	Receptor-mediated	CT can be propagated and transmitted through exosomes in the form of bioactivity. Under the action of HSP90 and PDI, CTA1 is transferred to the cytoplasm and absorbed during the formation of extracellular vesicles in the lumen	(Zanetti et al. 2018)
	Diphtheria Toxin	Receptor-mediated	Exosome directly induces toxin oligomerization on the membrane to protect cells	(Keller et al. 2020)
	Stx2	Vector of delivery	Stx2a exists on the surface of exosome. Exo-Stx2a is a new structure different from Stx2a. It originates from exosomes but does not exfoliate vesicles	(Watanabe-Takahashi et al. 2018)
	Stx2	Vector of delivery	Macrophages secrete exosome (Stx2-Exo) containing Stx2a, which induces a proinflammatory state and triggers cell death of receptor epithelial cells	(Lee et al. 2020)
	Staphylococcus aureus alpha toxin	Regulation of immune response	Exosome acts as bait to capture membrane virulence factors (such as porotoxins) to prevent target tissue damage	(Möller et al. 2021)
	Anthrax toxin	Vector of delivery	LF, the lethal factor of anthrax lethal toxin, can be transmitted from cell to cell through exosomes, which may play a toxic role over a long distance	(Abrami et al. 2013)
	Ochratoxin A (OTA)	Others	OTA-EXO triggers cell cycle arrest and induces changes in protein metabolism	(Zhu et al. 2021)

*TCS*: Trichosanthin; *CT*: Cholera toxin; Stx2: a second type of Shiga toxin; *OTA*: Ochratoxin A

### Receptor-mediated

**Fig. 3:**
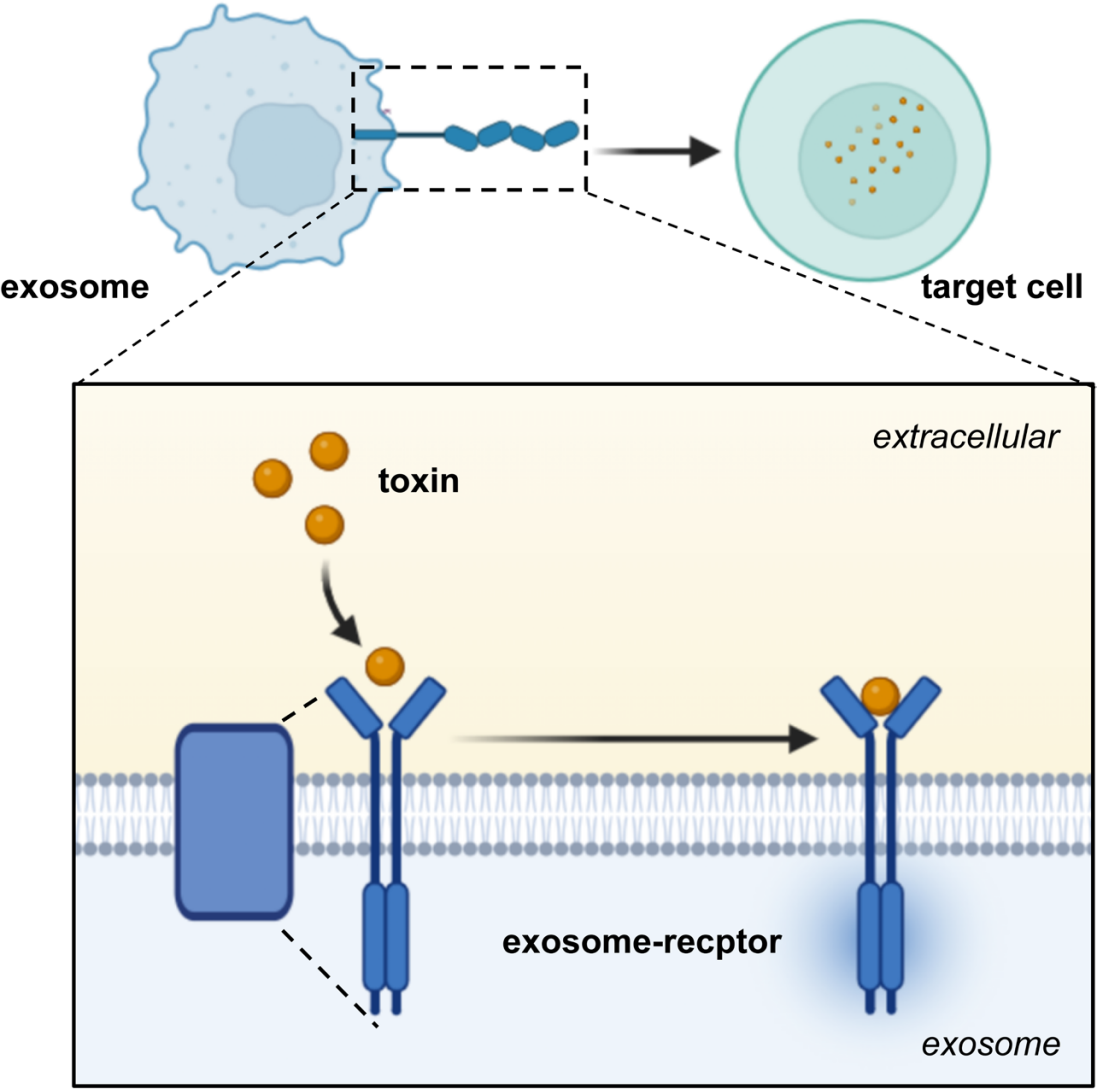
Exosomes act as receptors to mediate toxin toxicity. Toxins bind to exosomes and proliferate, while receptors on exosomes bind to target cells to cause cell damage

Exosomes directly activate receptors on the surface of target cells through protein molecules or lipid ligands, generate signaling complexes, and activate intracellular signaling pathways (Fig. 3). This effect will cause recipient cells to secrete more or less exosomes after stimulation after stimulation directly, thereby further expanding the intracellular influence. Cholera toxin (CT) activates the intracellular adenylate kinase of target cells directly through an exosome-mediated cell communication pathway. It has also been found that CT can be propagated in biologically active form through exosomes and then transferred in a typical endoplasmic reticulum retrograde pathway under the action of heat shock proteins 90 (HSP90) and protein co-enzyme protein disulfide isomerase (PDI). It can extend the pathophysiological effects from the initial host cells, such as the gut, to a variety of cells (Zanetti et al. 2018). When human A594 cells were exposed to diphtheria toxin, Autophagy-related (ATG) proteins reduced ADAM10 on the cell surface through lysosomal and proteasome-independent processes was observed. Consequently, the cells secreted more exosomes to protect themselves from diphtheria toxin by directly inducing toxin oligomerization on the membrane (Keller et al. 2020). Generally, this direct effect is applicable when the toxins have a protein structure or target specific cells.

### Vehicle of delivery

**Fig. 4:**
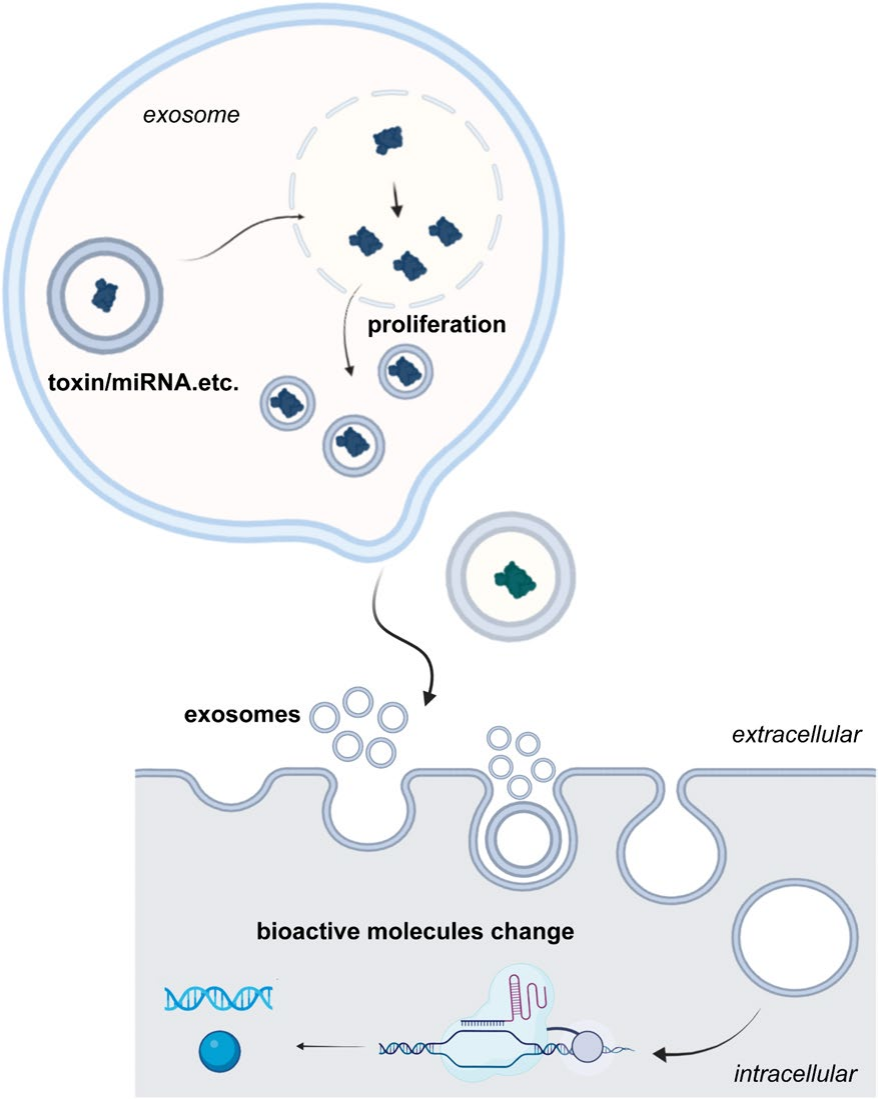
Exosomes act as vehicle of delivery. After the toxin attacks the cells, the active substances contained in the exosomes change and are further delivered to the target cells, causing changes in the active molecules

Exosomes can blend with the plasma membrane of cells or endocytose directly into the cells, carrying proteins, lipids, and other active molecules into the cells, thereby regulating the function and biological behavior of cells (Fig. 4). When exposed to external stimuli, exosomes deliver small RNAs to play a great concern role in reducing endotoxin and achieving homeostasis in the body. Alexander et al. (Alexander et al. 2015) injected exosomes containing miR-146a and miR-155 into LPS-treated mice and found that these miRNAs were delivered to various tissues, where they inhibited inflammatory-related gene targets and regulated inflammatory responses to endotoxin. circANTXR1 exists in the exosomes of liver cancer cells exposed to toxins. Exosome circANTXR1 overexpression promotes HCCLM3 cell proliferation and metastasis. Also, exosomes are participated in the intercellular transport process of circANTXR1, thereby affecting the biological function of HCC cells (Huang et al. 2021).

Exosomes have the potential as carriers to facilitate signal transduction between toxins. Since most exosomes are secreted by cells, exosomes change accordingly with biotoxins-induced endotoxin and cell apoptosis (Noonin and Thongboonkerd 2021). Exosomes from Stx2 (a second type of Shiga toxin)-treated THP-1 cells contained Stx2 and were phagocytosed by Gb3-positive cells, in which process they selectively induced Gb3-dependent apoptosis associated with Caspase3/7 activation. At the same time, the secreted exosomes Stx2-Exo contain a large amount of Exo-mRNA encoding inflammatory cytokines IL-1β, which aggravate the pro-inflammatory state of the receptor cells. Also, Stx2-Exo affects biomarkers concerning endoplasmic reticulum stress and MAPK pathway (Lee et al. 2020).

### Regulation of immune response

**Fig. 5:**
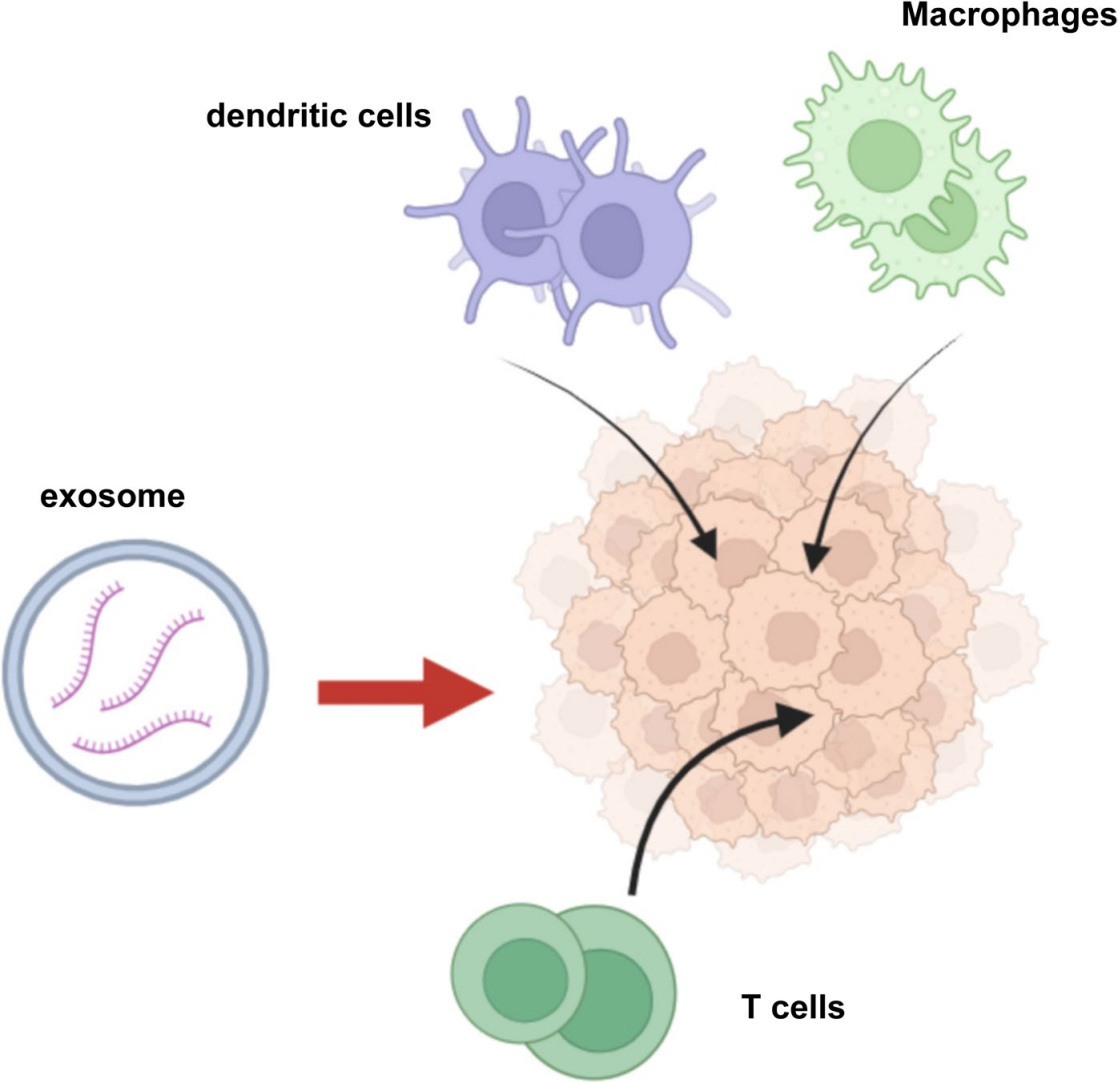
Exosomes act as regulatory factor of immune response. After toxins attack non-immune cells, exosomes communicate between cells and recruit immune cells such as macrophages, T cells, and dendritic cells for repair

The role of exosomes in immune regulation can be summarized as antigen presentation, regulation of host immune response, expression of some activation molecules or complement factors to cause immune surveillance, enhance tumor cell invasion, and mediate intercellular communication (Shao et al. 2020) (Fig. 5). Toxin-induced interactions in vivo involve complex biological processes. The presence of toxins can stimulate cells to produce exosomes, which are enriched in inflammation-related signaling molecules, or to recruit surrounding signaling molecules such as cytokines and chemokines (MHC-I and MHC-II). When they are taken up by adjacent or distant cells, they can activate or enhance the inflammatory response of these cells, thereby promoting the body's immune response to the toxin. Colchicine prevents the migrations of immune cells by inhibiting intracellular proinflammatory cytokines, vesicle trafficking, and exosomes release at the site of inflammation (Jerschke et al. 2023). Generally, various immune cells (e.g. dendritic cells, lymphocytes) have been expounded to release immunomodulatory exosomes. βγ-CAT isolated from the skin secretions of B.maxima (a frog species) stimulates the production and release of functional exosomes in mice, enhances the antigen-presenting ability of mouse bone marrow-derived immune cells, and activates T cell responses by secreting exosomes. Furthermore, these findings indicated that βγ-CAT transport membrane integration proteins and immune factors (MHC-I and MHC-II) between cells through vesicle transportation and the release of exosomes (Deng et al. 2020). Besides, exosomes exhibit unique, innate immune responses against bacterial infection as bait to entrap membrane-acting virulence factors, such as pore-making toxins, and protect target tissues (Sun et al. 2019). These "deplastids" act similarly to engineered exosomes, which neutralize toxins from Gram-positive bacteria. In addition, ATG proteins modulate Gram-positive bacteria-induced exosome production during host defense. This mechanism is different from the previously reported role of ATG16L1 in promoting plasma membrane repair during *Listeria monocytogenes* infection (Keller et al. 2020). Another interesting virulence mechanism was proposed by identifying important immune factors (IL6, caspase-1, IL-1b, IL-18, and IL-33) in exosomes released by EBV-infected cells (Sun et al. 2018b). In the innate immune sensor STING (IFN gene stimulator), miRNA and mRNA are exported by exosomes, which are then delivered to uninfected cells (Wang et al. 2023). The specific mechanism and functional significance of this process are currently unclear and require further study.

It can be concluded that exosomes play a dual role in the regulation of the immune system through either the toxin-induced body responses, which act as host programs to induce innate and adaptive immunity, or as evading strategies to eliminate inflammation (Wu et al. 2018).

## Conclusion and prospect

Exosomes are critical vehicles of biotoxins. They promote toxin infection and host immunity in terms of toxicity mechanism (Meldolesi 2018). Exosomes are derived from various cell species and contain multiple contents, which endow them with heterogeneity, providing sufficient biological functions and space for artificial transformation (Kimiz-Gebologlu and Oncel 2022). This paper reviews the role of exosomes as a medium in the toxicological mechanism of biotoxins. The role of exosomes in microbial toxins has been studied by many people, and animal toxins and phytotoxins are slightly involved (Bignell et al. 2018; Gao et al. 2021). Nevertheless, there are still many unanswered questions including (1) the role of exosomes in marine toxins remains to be explored, (2) there is no close connection between exosomes and general mechanisms such as oxidative stress caused by toxins, (3) the question of whether exosomes play different roles after exposure to different doses of the toxin and what the consequences of their expression are still to be addressed (Table 2). In order to make exosomes widely used in the field of biotoxins and make progress as much as possible.

**Table 2:** Difficulties of exosomes in the mechanism of toxin

	Difficulty	Reference
Exosome	Difficulty in extraction	(Lai et al. 2022)
	The morphology is similar to that of some microorganisms, and the identification is easy to be mixed	(Im et al. 2015)
	The application of exosomes has limitations	(Ludwig et al. 2019; Yang et al. 2019)
Toxins	A wide range of toxins	(Navale et al. 2021)
	The effects of exposure to different toxin doses vary	(Parak et al. 2024)
	The combination of multiple toxins has a wide range of toxicity	(Alengebawy et al. 2021)

At present, the difficulty of research on exosomes is still in the aspect of extraction (Wu et al. 2019). The understanding of exosome physiology, diversity, internalization and molecular cargo transportation is still very limited, and the extraction is limited to ultracentrifugation and other methods (Ludwig et al. 2019; Pegtel and Gould 2019). A series of problems still need to be solved. For example, the diameter of exosomes is similar to that of some fungal bacteria. Fungi and other microorganisms are inevitable in biotoxin research, and it is difficult to exclude the contamination of microbial particles by differential ultracentrifugation (Sadeghi et al. 2023). However, the combination of polyethylene glycol treatment and ultracentrifugation fulfills the need to purify exosomes from viruses, they can also be applied to the study of exosome-mediated biotoxins. Future research should consider the potential effects of more carefully, for example, whether the secretion process of the exosome is the same as that of ordinary cells, and whether other metabolites will be produced, whether the exosome will transmit signals as a carrier in the process of detoxification and detoxification of the toxin still needs to be further studied. Because exosomes can be secreted by blood, urine and other body fluids, they provide a non-invasive detection possibility. In the early stage of toxin exposure, the health level of the body can be judged by detecting the content of exosomes in blood and urine or the level of special markers, which is of great significance for improving the acceptance of patients and the convenience of detection. However, at present, these applications exist in the development of diseases, and their applications in toxins are less, which are worthy of further development. With the development of isolation and purification technology, the focus has gradually shifted from lab research to clinical application. We believe that exosomes can contribute to establishing novel diagnosis and detoxification approaches for biotoxins.

## Data Availability

Data availability is not applicable to this article as no new data were created or analyzed in this study. The data that support the findings of this study are available from the corresponding author, Xiaoyun He, Kunlun Huang (Email: hexiaoyun@cau.edu.cn; foodsafety66@cau.edu.cn), upon reasonable request.
